# Interventions to improve discharge from acute adult mental health inpatient care to the community: systematic review and narrative synthesis

**DOI:** 10.1186/s12913-019-4658-0

**Published:** 2019-11-25

**Authors:** Natasha Tyler, Nicola Wright, Justin Waring

**Affiliations:** 1 NIHR Greater Manchester Patient Safety Translational Research Centre, The University of Manchester, Manchester, UK; 2 School of Health Sciences, University of Nottingham, Nottingham, UK; 3 Health Services Management Centre, School of Social Policy, University of Birmingham, Birmingham, UK

**Keywords:** Systematic review, Care transitions, Mental health, Interventions, Discharge, Acute services, Psychiatric discharge, Hospital discharge

## Abstract

**Background:**

The transition from acute mental health inpatient to community care is often a vulnerable period in the pathway, where people can experience additional risks and anxiety. Researchers globally have developed and tested a number of interventions that aim to improve continuity of care and safety in these transitions. However, there has been little attempt to compare and contrast the interventions and specify the variety of safety threats they attempt to resolve.

**Methods:**

The study aimed to identify the evidence base for interventions to support continuity of care and safety in the transition from acute mental health inpatient to community services at the point of discharge. Electronic Databases including PsycINFO, MEDLINE, Embase, HMIC, CINAHL, IBSS, Cochrane Library Trials, ASSIA, Web of Science and Scopus, were searched between 2000 and May 2018. Peer reviewed papers were eligible for inclusion if they addressed adults admitted to an acute inpatient mental health ward and reported on health interventions relating to discharge from the acute ward to the community. The results were analysed using a narrative synthesis technique.

**Results:**

The total number of papers from which data were extracted was 45. The review found various interventions implemented across continents, addressing problems related to different aspects of discharge. Some interventions followed a distinct named approach (i.e. Critical Time Intervention, Transitional Discharge Model), others were grouped based on key components (i.e. peer support, pharmacist involvement). The primary problems interventions looked to address were reducing readmission, improving wellbeing, reducing homelessness, improving treatment adherence, accelerating discharge, reducing suicide. The 69 outcomes reported across studies were heterogeneous, meaning it was difficult to conduct comparative quantitative meta-analysis or synthesis.

**Conclusions:**

The interventions reviewed are spread across a spectrum ranging from addressing a single problem within a single agency with a single solution, to multiple solutions addressing multi-agency problems. We recommend that future research attempts to improve homogeneity in outcome reporting.

## Background

The transition from acute mental health inpatient to community care is often a vulnerable period in the pathway, where people can experience additional risks to their mental health and psychological wellbeing. Previous research with service users has found discharge to be a chaotic, stressful and emotionally charged time [1]. The term “revolving door” is widely used to describe how mental health service users can repeatedly transition between hospital and community care, and then back into hospital within a very short timeframe. However, the terminology of “revolving door”, within this context, has been criticised by service users and the survivor movement for situating the problem of repeated transitions with the individual rather than with the systems around them [2]. This ‘circuit of care’ stems not only from the person’s underlying health conditions, but often from the challenges of ensuring the continuity of care following inpatient discharge. A pilot qualitative study conducted in the United Kingdom (UK) [1, 3] identified examples of these challenges including; (1) problems with medication management and maintaining concordance; (2) increased risk to self (i.e. suicide) and others (particularly family members); (3) poor information sharing between services leading to both gaps and duplication in provision; and (4) poorer mental health due to the distress caused by multiple often difficult transitions. In recent years there has been considerable research evidencing the relationship between discharge from acute mental health inpatient units and suicide [4]. Research shows, for example, that between 2005 and 2015 17% of people who completed suicide had recently been discharged from acute hospital services [4]. The significance of suicide as a marker of quality during and after acute care is further indicated by its routine use in many studies and evaluations of interventions to support hospital discharge, alongside other measures such as readmission and length of stay [5–8]. However, the variety of challenges that are present at this sensitive time in the service user journey transverse far beyond what can be measured solely using readmission or death by suicide rates.

Researchers globally have developed and tested a number of interventions that aim to improve the care transition. Some interventions are targeted to a particular group, i.e. to reduce the risk of post-discharge homelessness [9, 10]. Other interventions focus on a particular source of risk to health following discharge, such as medicines management [11, 12]. Whilst others are concerned with coordinating care, more broadly, between different agencies [13, 14].

How such interventions are configured, in terms of their cause-and-effect mechanisms, and implemented in different contexts provides additional insight about how service leaders and researchers understand and seek to address the problems of care transitions from acute mental health settings. That is, whether the source of risk is located within the individual who needs additional education or support, with the care system in terms of the problems of coordinating care, or with wider social and community factors. As such interventions are so varied, it shows that different groups articulate the challenges associated with discharge differently. It is increasingly recognised that the evaluation of quality improvement interventions, such as those for hospital discharge, should more explicitly articulate and appraise the underpinning theory of change for a given intervention (the rationale and assumptions about mechanisms that links processes and inputs to outcomes, also specifying the conditions necessary for effectiveness) [15–17].

There has been little attempt to compare and contrast the interventions and specify the variety of problems they implicitly or explicitly attempt to resolve. Previous systematic reviews of discharge interventions have been restrictive. For example, one systematic review focused only on transitional interventions that aimed to reduce readmission [18]. Another review was restricted to interventions that were delivered pre-discharge [19]. The problems each intervention hopes to address are often varied or implicit, as is the study design and outcome measures used. There has been little attempt to descriptively compare the types of interventions that exist and the quality and safety challenges that they aim to address. By removing the search restrictions we hope to compare and contrast the interventions that have been tested and look for commonalities and differences in effectiveness and in the way different researchers articulate the problems associated with discharge.

### Aim

To identify and synthesise the evidence base for interventions to support continuity of care and safety in the transition from adult acute mental health inpatient service to community care services at the point of discharge.

## Methods

### Study design

Systematic review. The review follows Preferred Reporting Items for Systematic Reviews and Meta-Analyses (PRISMA) reporting guidelines. For the PRISMA checklist, see Additional file 1. The study protocol was prospectively registered with PROSPERO (CRD42018097475).

### Data sources

Medical and social science databases (including PsycINFO, MEDLINE, Embase, HMIC, CINAHL, IBSS, Cochrane Library Trials, ASSIA, Web of Science and Scopus) were searched from 1st January 2000 to May 2018. A combination of controlled vocabulary index and free text terms were used to search electronic databases, including terms relating to discharge (e.g. “discharge”, “hospital discharge”, “psychiatric hospital discharge”, “transfer”), mental health (e.g. “exp mental health”, “mental* disorder*”, “mental illness*”, “schizophr*”, “suicid*”), and interventions (e.g. “exp intervention”, “discharge intervention”, “discharge planning) see Additional file 2 for a full list of search terms used. Where a controlled vocabulary index did not exist for a database or website, only free text terms were used. Forward and backward searches were conducted on included papers using Google Scholar. All identified references were imported to Mendeley.

### Selection criteria

#### Inclusion criteria

Papers were eligible for inclusion if they 1) addressed adults (18–65); 2) admitted to an acute inpatient mental health setting; 3) functional conditions (mental disorders other than dementia, and includes severe mental illness such as schizophrenia) 4) reported on health interventions or service provision; 5) interventions that aimed to improve discharge from the acute ward to the community. Papers were eligible for inclusion in the review if they were peer-reviewed, empirical studies (quantitative or qualitative design) and reported original data. We included interventions that aimed to improve the transition from in-patient to community care for an adult population. Components of the intervention could be delivered prior to discharge, shortly after discharge or could span both. Papers were not excluded based on country of origin.

#### Exclusion criteria

We excluded interventions not related to functional adult mental health (i.e. medical, surgical, paediatric/older adults and organic conditions) and that were involuntary in nature (e.g. involuntary treatment orders or forensic interventions). This is because we were interested in clinical interventions that promoted safety at discharge rather than the use of legislation to forcibly transition someone from one setting to another. We excluded interventions focused on treating specific psychiatric disorders (for example, medication or specific psychotherapies) unless there was a component that specifically focused on improving the transition from in-patient to community care. Theses, editorials and opinion pieces were excluded. Studies that did not include primary data were removed (e.g. systematic reviews). If a research team reported the same study in multiple papers, only the original paper was included. The search was restricted to English language only papers.

### Screening

All papers identified by the database searches were downloaded to Mendeley to remove duplicates and screened at the title/abstract level for inclusion in full-text review by one reviewer (NT). Two reviewers (NW and JW) each independently screened 20% of the titles and abstracts. Group discussions resolved any differences between the reviewers and confirmed the final included studies list.. Details of the excluded papers and reasons for exclusion are available on request.

### Data extraction and quality appraisal

Data were extracted from each study into a standardised table. Data extraction was conducted by one researcher NT. As a check, one reviewer (NW) checked the extracted data from a random 10 papers. Data were extracted related to: a) Aim of study b) Disciplinary perspective c) Theoretical background d) Geographical context e) Context for study f) Method g) Sample h) Analytical approach i) Outcomes measured j) Intervention details k) Evidence of outcome/Effect of intervention l) Evidence about genesis.

The methodological quality of studies was independently assessed by one reviewer (NT) using the Mixed Methods Appraisal Tool (MMAT) [20]. Complete listings of all studies and quality appraisal scores are presented in Additional file 3.

### Analysis

Due to the heterogeneity in the data and outcomes reported, statistical pooling of the data was not used. Therefore, a narrative approach to synthesising the included studies was taken. Narrative synthesis is an approach to systematic review and synthesis of findings from multiple studies that depends primarily on using words and text to explain and summarise the findings [21]. The narrative synthesis was conducted using guidance, by one reviewer (NT) [22]. A four stage process was followed; 1) developing a theory of how the intervention works, why and for whom; 2) developing a preliminary synthesis; 3) exploring the relationships between and within the studies; and 4) assessing the robustness of the synthesis. Additional file 4 illustrates the synthesis process and the tools used. Extracted data were analysed by one reviewer (NT) using narrative synthesis.

## Results

### Data sources

**Fig. 1:**
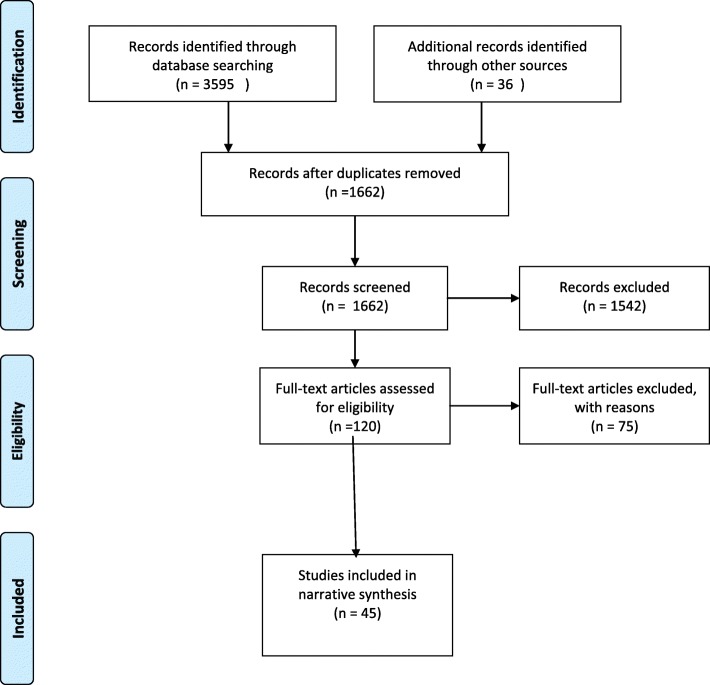
PRISMA flow chart. PRISMA flow chart to report numbers of included and excluded papers at each stage

The search of the electronic databases generated 3595 hits including duplicates. Citation mapping revealed a further 36 papers which were included. One thousand six hundred sixty-two unique papers were identified; 1542 papers were excluded after screening and 120 full papers were reviewed. We excluded 75 full texts for the following reasons: (1) not related to mental health, (2) not related to an adult populations, (3) not describing an intervention (4) not including primary empirical results, (5) not an acute inpatient population (i.e. forensic or organic) (6) not focused on discharge. Therefore, the total number of papers from which data were extracted was 45 (Fig. 1). Table 1 presents the 45 studies included in the review, alphabetised by author name, location, intervention, population, method, findings and the problems they aim to address. We first of all grouped the studies in terms of design and populations. We then grouped the studies by the type of interventions, then the problems they aim to address. Finally, we collate the outcomes reported and the facilitators and challenges reported.

**Table 1:** Tabulation and description of the studies used in the systematic review

ID	Authors and year	Location and setting	Intervention	Participants	Method	Main findings	Main aim/ problem to address
1	Abraham et al. (2017)	USA, 1 urban psychiatric hospital	Pharmacist involvement to improve care co-ordination	16 health professionals, 6 patients, 20 patient charts (SMI patients)	Evaluation- interviews and observations of charts	Increased pharmacist involvement in LAI care coordination may contribute to bridging gaps in medication adherence to optimize treatment outcomes.	To support long-term medication adherence and patient outcomes
2	Attfield et al. (2017)	UK, 2 trusts	Diagnostic-driven Integrated Care Pathways (ICPS)	A random sample of 400 service users	Retrospective case comparison study	The ICP Trust had a 13.5 day shorter average length of stay, (statistically significant). No significant differences in readmission or 7-day follow-up.	Reducing unnecessary tests, interventions and duplication within the care process
3	Bauer et al. (2012)	Germany, 1 hospital	SMS-based maintenance intervention	165 females. Eating disorders	RCT	Somewhat significant difference in readmission (depending on analysis). Significant difference in treatment utilisation.	Maintain treatment
4	Bennewith et al. (2014)	UK, 3 inpatient wards in southwest England, mixed urban/rural	A contact-based intervention for people recently discharged (letters sent to sus)	102 patients received a letter, 45 received all letters	Pilot case study. Interviews, analysis of outcomes (readmission)	Post-discharge, qualitative interviews with service users showed that most already felt adequately supported and the intervention added little to this.	To reduce suicide post-discharge by providing social connectedness
5	Bonsack et al. (2016)	Switzerland, 1 psychiatric hospital	Transitional case management	51 intervention, 51 control	RCT	Increased short-term rate of engagement with ambulatory care despite no differences between the two groups after 3 months of follow-up. Intervention did not significantly reduce the rate of readmissions during the first year following discharge.	Improve engagement with care, reduce readmission
6	Botha et al. (2018)	South Africa, 1 hospital	90-day transitional care intervention (four phone calls and one home visit, focusing on maintaining adherence, appointment reminders and psychoeducation.)	60 male patients	Retrospective comparison to matched control group	No effect on readmission rates in this setting.	Bridge gap, reduce readmissions
7	Chen (2014)	USA, all of the community agencies providing CTI in NYC (4)	Community support in critical time intervention (CTI)a time-limited, short-term psychosocial rehabilitation. Program designed to facilitate the critical transition from institutional to community settings	12 CTI workers	Interviews	CTI workers self-identified as “extra support” to develop community ties that will help clients sustain stable housing. Propose a transient triangular relationship model, involving three dyadic relationships (worker-client, worker-primary support, primary support client).	To facilitate effective transitional services and enhance continuity of care. Breaking the vicious cycle between institutionalization and homelessness
8	D’Souza (2002)	Australia, rural hospital	Telemedicine (psycho-educational programme and MDT videoconferencing post-discharge)	51 (24 intervention, 27 control) male and female	Controlled study	More side effects in control group, more treatment adherence and satisfaction in intervention group.	Improve treatment adherence
9	De Leo and Heller (2007)	Australia, 1 psychiatric inpatient unit	Intensive case management follow up of high risk people (ICM was weekly face-to-face contact with community case manager and telephone calls from counsellors)	60 male service users with a history of suicide attempts	RCT (TAU or intervention)	People in ICM had lower depression scores, suicidal ideation, QoL, more contact with services, better relationships with therapists and were satisfied with service.	A solution to the reduced care following discharge that is linked to suicide.
10	Exbrayat et al. (2017)	France, single centre	Telephone follow-up 8,30 and 60 days post attempted suicide	436 patients (387 control patients who were matched from pre-intervention records)	Controlled study	Very early telephone follow-up of our patients effectively reduced recidivism and seemed to be the only protective factor against repeated suicide attempt.	To reduce suicide attempts post-discharge
11	Forchuk et al. (2005)	Canada, 26 wards, 4 hospitals	Transitional discharge model (TDM)	390	Randomised clinical trial using a cluster design	Costs and quality of life were not significantly improved post-discharge compared with the control group. Although not predicted a priori, intervention subjects were discharged an average of 116 days earlier per person.	Reduce bed occupancy, improve quality of life
12	Forchuk et al. (2008)	Canada, 1 hospital	Intervention to prevent homelessness- immediate assistance in accessing housing and assistance in paying their first and last month’s rent	14 participants at risk of being discharged without housing (7 in intervention group)	RCT, incl. interviews	All intervention group maintained housing after 3 and 6 months. All but one individual in the control group remained homeless after 3 and 6 months. Results of this pilot were so dramatic that randomizing to the control group was discontinued	To reduce discharge from inpatient wards to shelters or the street
13	Forchuk et al. (2012)	Canada, 6 hospitals	Transitional relationship model (TRM) (providing hospital staff involvement until a therapeutic relationship has been established with a community care provider as well as peer support.)	No participant numbers as ethnographic analysis. 14 *A* wards, 12 *B* wards and 10 *C* wards.	A quasi-experimental, action-oriented research design	Staged large-scale implementation allowed for iterative improvements to the model leading to positive outcomes. This study highlights the need to address work environment issues, particularly inter-professional teams.	To improve staff uptake of interventions
14	Forchuk et al. (2013)	Canada, all patients in Ontario at risk of homelessness, 1 acute care hospital, 1 territory	Intervention to prevent homelessness - Pre-discharge assistance in securing housing	112 men and 107 women at risk of homelessness post-discharge	Programme evaluation design- interviews, focus groups	The results highlight several benefits of the intervention and show that homelessness can be reduced by connecting housing support, income support, and psychiatric care.	To stop people being discharged to street or shelters
15	Ghadiri Vasfi et al. (2015)	Iran, 1 hospital	Aftercare Services (three components: follow-up Care (home visits or telephone follow-up), family psychoeducation, And social skills training for patients.)	120 patients (schizophrenia and bipolar) ages 15–65. 60 control	RCT	The cumulative number of hospitalizations during the follow-up period was 55 for the control group and 26 for the intervention group. Length of stay was significantly greater in the control group. Psychopathology was significantly less severe in intervention group compared with the control	Reducing readmissions and length of stay
16	Hampson et al. (2000)	UK, 1 trust (North Nottingham and Hucknall)	Community Link Team (CLT) to facilitate early discharge- team-based service offering intensive support during the day	142 (all admissions to team in 12 month period)	Retrospective comparison	Median length of stay during CLT project was 19 days, a highly significant reduction from 36 days in the NABUS study. Cannot be attributed to team but justifies a RCT to test this hypothesis,	To speed up discharge due to costs to provider and patients
17	Hanrahan et al. (2014)	USA, 1 hospital	Transitional care model (TCM)	40 (20 control)	RCT	The intervention group showed higher medical and psychiatric rehospitalisation than the control group. Emergency room use lower for intervention group but not statistically significant. Continuity of care with primary care appointments were significantly higher for the intervention group. The intervention group’s general health improved but was not significant	Reduce transition failures
18	Hegedus et al. (2018)	Switzerland, 2 wards, 1 hospital	Short transitional intervention in psychiatry (step)	14 control, 15 intervention	Quasi-experimental pilot study to determine the feasibility of the intervention,	The intervention did not affect primary or secondary outcomes; however, it was shown to be feasible, and patients’ feedback highlighted the importance of post-discharge contact sessions.	Prepare patients for situation outside of hospital
19	Hengartner et al. (2015)	Switzerland, 1 catchment area, which is an urban/suburban area of high-level resources near the city of Zurich	Post-discharge network coordination	3 patients	Case studies- narrative review and qualitative analysis of three patients who participated in the program	Case reports revealed that patients’ social networks are small and their relationships are commonly conflictual and unstable.	Reducing readmission, improving mental health and psychosocial functioning. Improve hospital discharge planning and to ease the transition
20	Hengartner et al. (2016)	Switzerland, 1 catchment area, which is an urban/suburban area of high-level resources near the city of Zurich	Post-discharge network coordination	151 patients	RCT using parallel group blocking	In the short-term, no significant effect emerged in any outcome. In the long term the two groups did not differ significantly with rate and duration of rehospitalisation. The intervention did not reduce psychiatric symptoms, did not improve social support, and did not improve quality of life.	Reducing readmission, improving mental health and psychosocial functioning. Improve hospital discharge planning and to ease the transition
21	Herman et al. (2011)	USA, 2 transitional residences in hospital grounds metropolitan area	Critical Time Intervention to prevent homelessness	150 previously homeless men and women with SMI	RCT	CTI group had less homelessness than TAU	Reduce homelessness following discharge
22	Jenson et al. (2010)	Canada, poor city, high unemployment, 1 acute ward and 1 community service provider within same region	Community-Based Discharge Planning (in-reach model- discharge planner based in community visits ward daily)	36 service users	Single group programme evaluation, analysis of admin data and interview with clients	Readmission rates were 40% lower in the year following the change in service delivery model. This change was statistically significant.	To shift mental health services from institution to community
23	Juven-Wetzler et al. (2012)	Israel, 1 ward	“Continuation of Care” model (continuing follow-up in the ward, by the same staff, instead of being referred to the outpatient department.)	35 service users	Pre and post within participant design	The number of hospitalizations in the 18 months following the index hospitalization was 1.79 _ 3.51 as compared to 4.67 _ 1.79 before the index hospitalization (*p* = 0.0002), and the number of days of hospitalization 18 months after was 24 _ 41.65 as compared to 119.71.	To reduce length of stay and readmission
24	Kariel-Lauer (2000)	Israel, 1 hospital	Re-entry group (short-term group meetings- psychoeducational approach)	75 participants (42 in intervention) men and women	A controlled study	Intervention group had less readmissions, high rates of absorption into therapy and remaining in therapy	Reduce hospitalisations, increase compliance with outpatient appointments
25	Kaspow and Rosenheck (2007)	USA, 8 veteran medical centres	Critical time intervention Case management (a modification of the critical time intervention (CTI) community case management model)	278 control cohort, 206 intervention cohort	Nonrandomized pre–post cohort design	19% more days housed in each 90-day reporting period over the one-year follow-up and 14% fewer days in institutional settings. Veterans In phase 2 also had 19% lower addiction severity index alcohol use scores,14% lower drug use scores And 8% lower psychiatric problem scores	Reduce homelessness,
26	Khaleghparast et al. (2013)	Iran, 2 hospitals	Discharge planning (self-care training programme/nursing process model)	46 service users	Longitudinal clinical trial	The intervention group had improved clinical symptoms and higher knowledge levels compared with control group. Statistically significantly lower readmissions in the intervention group.	To increase patient knowledge, reduce clinical symptoms and rehospitalisation.
27	Khanbhai et al. (2018)	Australia, 1 medical centre	Discharge checklist	230 checklists	Quasi-experimental, pre–post intervention design	There was a small, but statistically non-significant, reduction in readmission rates.	Reduce readmission
28	Kidd et al. (2016)	Canada, 1 large hospital in city	‘Welcome Basket.’ (6 week peer support, contact on wards, basket of items, environmental support)	23	Evaluation- a mixed methods design, pre-post for quantitative outcomes, interviews and readmission rates	Pre–post analysis indicated no change in psychiatric symptoms but improvement in community functioning, community integration, and quality of life. No difference in readmission	Reduce suboptimal outcomes in first month, bridge gap
29	Kisely et al. (2017)	Australia, 1 hospital- intervention and control wards within it	Motivational aftercare planning (motivational interviewing with advance directives)	100 intervention plans, 197 control, 20 service user interviews	Controlled before-and-after design, interviews	Intervention ward improved significantly (e.g. identification of triggers significantly increased from 52 to 94%, This did not occur in the control wards. Interviews showed improvements in experiences of discharge planning.	To increase patient input into discharge planning, increase treatment plan following
30	Lawn et al. (2008)	Australia, 3 hospitals	Peer support	No participant numbers in evaluation	Evaluation methodology.	Intervention at this stage of their recovery seems highly effective as an adjunct to mainstream mental health services. It has personal benefit to consumers and peers, substantial savings to systems, as well as much potential for encouraging mental health service culture and practice towards a greater recovery focus and improved collaboration with GPs	To reduce hospital avoidance and facilitate early discharge
31	Lin et al. (2018)	Taiwan, 1 hospital	Needs-oriented hospital discharge planning for caregivers	114 caregivers (of people with schizophrenia) 57 in each group	A quasi-experimental research design	The caregiver burden and health status of the experimental group improved more significantly compared with control group.	Reducing readmission and improving medication adherence, reducing care giver burden
32	Puschner et al. (2011)	Germany, 5 hospitals	Needs-oriented discharge planning intervention	491	Multicentre RCT	No effect of the intervention on outcomes.	Reduce high utilisation of inpatient care
33	Reynolds et al. (2004)	Scotland, 1 unit, 3 wards	Transitional Discharge Model (ward nurse worked with SU until relationship built with community nurse, then support from service users)	25 services user (14 control, 11 experimental)	Randomised experimental design	Both control and experimental group demonstrated significant improvements in symptom severity and functional ability after 5 months. Usual treatment subjects in the control group were more than twice as likely to be re-admitted to hospital.	Readmissions and not able to adapt to community, focus on need for relationships
34	Rose et al. (2007)	USA, 1 large urban medical centre, mostly African- American patients	Transitional care model a nurse-based in-home transitional care intervention	10 service users (schizophrenia, bipolar)	Evaluation- analysis of nurse logs	Offers an alternative to patients who might otherwise be left poorly treated or untreated in the community setting.	Lack of continuity of care and meet immediate post discharge needs of SU
35	Sato et al. (2012)	Japan, 5 hospitals	Community re-entry program. Discharge preparation program (psychosocial program for preparing long-term hospitalized patients)	26 intervention, 23 control (schizophrenia)	RCT	The program may be capable of promoting discharge of long-term hospitalized psychiatric patients. There was no significant difference between both groups for number of patients discharged 6 months after end of program.	To reduce length of stay
36	Scanlan et al. (2017)	Australia, 3 geographical areas, large non-governmental mental health service	Peer-delivered, transitional and post-discharge support program	38 service users	Evaluation, outcome measures, interviews	Participants reported improvements in functional and clinical recovery and in the areas of intellectual, social and psychological wellness. Self-report of hospital readmissions suggested that there was a reduction in hospital bed days following the program	Reduce readmission, increase wellbeing
37	Shaffer et al. (2015)	USA, 6 community-based provider organizations within network of a not-for-profit, managed behavioural health care organization	Brief critical time intervention (a brief, three-month version of CTI)	149 adults with readmission within 30 days, 224 control	A quasi-experimental investigation	BCTI was associated with decreased early readmission rates,	Reduce readmission
38	Shaw et al. (2000)	Scotland, 3 acute wards, 1 hospital	Pharmacy discharge planning (receiving a baseline Pharmaceutical needs assessment, information about medicines and then a Pharmacy discharge plan sent to their community pharmacy)	97 service users	Controlled study	No significant difference between the groups in baseline medicine knowledge. One week post-discharge, both groups showed Significant improvement in knowledge of medication from baseline and was maintained at 12 weeks. Fewer medication problems for the intervention group.	To reduce medicine-related problems that cause readmission
39	Simpson et al. (2014)	UK, 4 wards, inner city (London)	Peer support	46 service users 23 peer support 23 control	Pilot randomised controlled trial with economic evaluation	No statistically significant benefits for peer support for hope or QoL, there is an indication that hope may be further increased in those in receipt of peer support. The total cost per case for the peer support was £2154 compared to £1922 for control.	To increase hope and quality of life
40	Smelson et al. (2010)	USA, 1 acute inpatient psychiatric unit	Brief Treatment Engagement (5 h per week of services- assertive community treatment using BCTI, peer support, dual recovery therapy)	102 veterans, (56 control)	Prospective randomized trial	69% Of intervention participants attended an outpatient appointment within 14 days of discharge, compared to only 33% of control. Intervention participants were also significantly more likely to be engaged in outpatient services at the end of the intervention period.	Treatment engagement
41	Taylor et al. (2014)	USA, 1 large psychiatric hospital	Brief care management Intervention (brief interview prior to discharge)	87 intervention, 108 control, 195 total	Controlled study	Individuals in the control group were more likely to be readmitted within 30 days of an index readmission than individuals in the intervention group.	Increase aftercare engagement, reduce readmissions
42	Tomita et al. (2012)	USA, 2 New York City hospitals	Critical time intervention (CTI)	150 total previously homeless men and women	RCT	At the end of the follow-up period, psychiatric re-hospitalization was significantly lower for the group assigned to CTI compared with the usual services group.	Reducing readmission
43	Virgolesi et al. (2017)	Italy, 3 hospitals in Rome	Nursing discharge programme (a short-term nursing discharge programme with follow-up phone calls 7–10 days)	135 patients	Prospective correlational design	The interpersonal and educational nursing intervention improves adherence to a treatment plan.	Medication adherence and patient satisfaction
44	Walker et al. (2000)	UK, 3 wards (2 control)	Discharge co-ordinators	343, 119 intervention, 224 control	Controlled cohort study	No differences in outcomes (readmission, LoS, mental health status, satisfaction). More satisfaction for those without intervention	Improve communication between primary and secondary care
45	Zheng and Arthur (2005)	China, 1 large hospital in Beijing	Family education	101 patients (schizophrenia)	RCT pre-test, post-test	Significant improvement in knowledge about Schizophrenia in the experimental group. Significant difference in symptom scores and functioning at 9 months after discharge.	Knowledge about condition and rehospitalisation. There is a need for culturally sensitive family treatments offered by nurses

### Study design and population

**Fig. 2:**
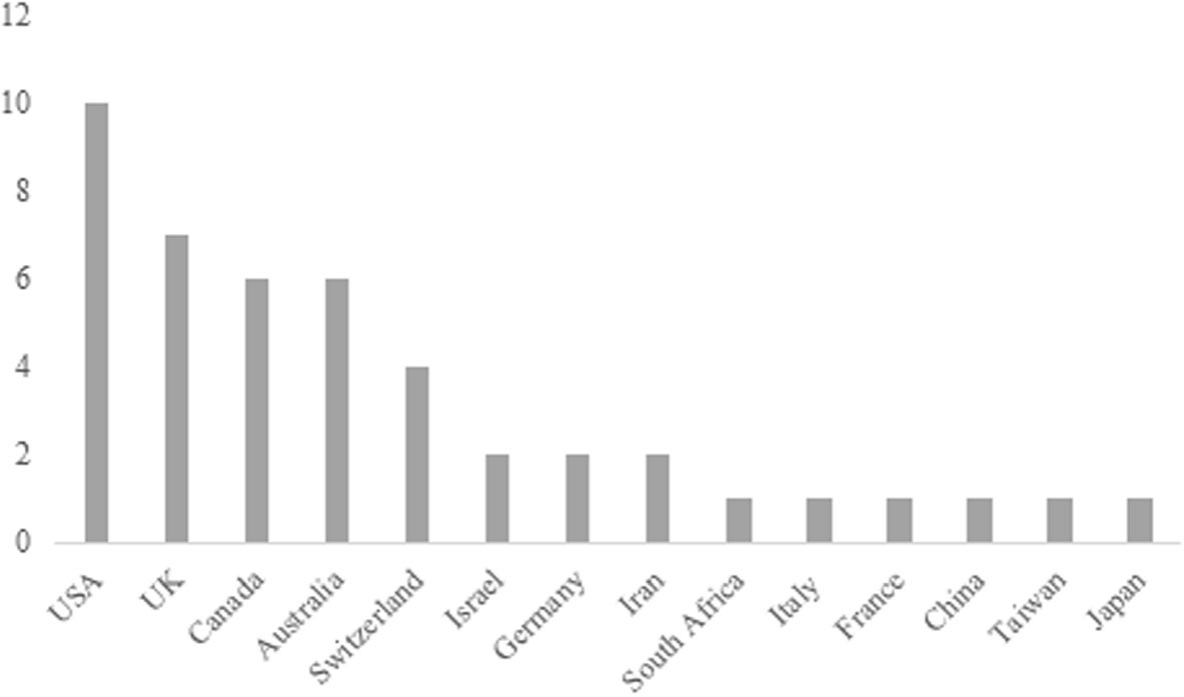
Countries of origin: The countries of origin of the papers included in the systematic review

The majority of the studies included in the review were conducted in high income countries, primarily the United States of America (USA), UK, Canada and Australia. However, a limited number were conducted in low and middle-income countries, such as Iran and China (see Fig. 2).

**Fig. 3:**
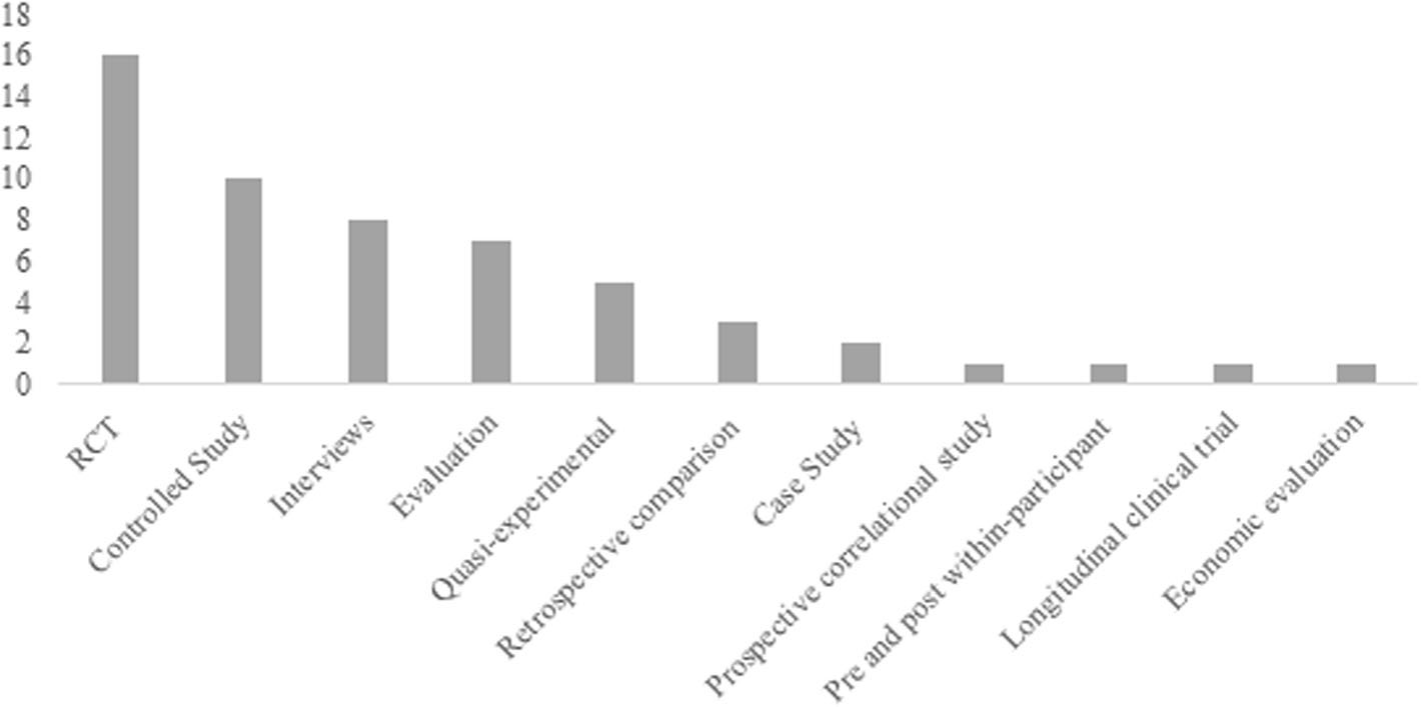
Design: The design of the papers included in the systematic review

The methodological design used in the papers varied considerably, the most common design used was a randomised controlled trial; which is often considered the highest quality research [23]. However, many of the papers were evaluation studies, often a mixed methods review of the effectiveness of a pilot or controlled studies using a non-randomised comparison group, see Fig. 3.

Given the variation in design, there was also variation in the number of participants recruited in each study. Whilst most studies contained more than 20 participants (93%), the majority contained less than 200 (78%). Only 7% of studies had more than 400 participants. It must be noted that the participant numbers often included the control group; in which case only half of the participant group received the intervention.

### Types of interventions

During the preliminary synthesis we used the clustering method to identify similarities between interventions (Table 2). Table 2 highlights this clustering, for some interventions this was explicit due to the use of a name for a distinct approach (i.e. Critical Time Intervention, Transitional Discharge Model) for others this involved implicitly grouping interventions based on key components (i.e. peer support, pharmacist involvement).

**Table 2:** Arbitrary clustering of interventions based on intervention categories

Intervention category	No of papers	Authors name and years	Description of intervention
Critical Time Intervention	5	Tomita (2014), Kasprow (2014), Herman (2011), Shaffer (2015), Chen (2014)	• Focused on homelessness • Delivered by CTI workers • Develop therapeutic relationship • Time limited (gradually reduced contact), Small caseloads, Community based • Phase 1: Transition: Provide support & begin to connect client to people and agencies that will assume the primary role of support • Phase 2: Try-Out: Monitor and strengthen support network and client’s skills. • Phase 3: Transfer of Care: Terminate CTI services with support network safely in place. • Also includes Brief CTI (shorter time)
Transitional Discharge Model (TDM)	3	Forchuk (2005), (2012), Reynolds (2004)	• Ward nurses work with SU until a therapeutic relationship is established with the community worker • Then peer support introduced • AKA/similar to Transitional relationship model (TRM)
Transitional care model	2	Hanrahan (2014), Rose (2007)	• Nurse based in home transitional care intervention, to increase CoC • A) comprehensive discharge planning • B) home visits and telephone contacts with a nurse (assessments, care, psychoeducation) • Aimed at most challenging patients with long history of readmission • Immediately providing intensive support and identifying problems early before readmission • Increase QoL through symptom management, medication adherence and enhanced family support
Peer Support	3	Lawn (2005), Scanlon (2017), Simpson (2014)	• Scanlan: Peer-delivered support programme: peers delivering providing individualised practical and emotional support to individuals for 6–8 weeks following discharge • Simpson: Peer support workers to provide peer support for 4 weeks to discharged service users, initial contact begins on ward • Lawn: Peer support workers trained alongside other health professionals. Service users matched to peers experience and skills, 8–12 h, 1–2 week period. Also hospital avoidance packages for those who are thought to need them.
Contact based interventions	6	Bennewith (2014), Bauer (2012), D’Souza (2002), Exbrayat (2017), De Leo (2007), Botha (2018)	• Bennewith: Letters sent to follow up recently discharged service users at home • Bauer: SMS sent to recently discharged service users about maintaining treatment • D’Souza: MDT videoconferencing with rural patients post-discharge • Exbrayat: Nurse telephone follow up 8, 30 and 60 days post suicide attempt • De Leo: Intensive case management – Weekly face-to-face contact with community case manager and telephone calls from counsellors • Botha: 90 Day Transitional care intervention – 4 phone calls, 1 home visit focusing on maintaining adherence, appointment reminders and psychoeducation
Role-based Interventions	6	Walker (2000), Virgolesi (2017), Hengartner (2016,17), Jenson (2010), Bonsack (2016), Hampson (2000)	• Walker: Discharge co-ordinators – educational role with service users and family, develop relationships, 6–8 weeks post-discharge, Dr. routinely telephoning GP practice regarding impending discharge and arrange an appointment with GP within 7 days of discharge, posting discharge summary to practice • Virgolesi: Nursing discharge programme- information interventions provided by nursing staff, direct hospital medication, distribution and follow-up telephone calls. Nurses attend a 1-h class organised into 5 modules: introduction to medication adherence, conceptual framework of medication adherence, medication adherence, intervention programmes, structure of medication adherence interview, and case studies. • Hengartner: Post-discharge network co-ordinator – delivered by social workers, support service users to build and maintain social network and link to community care system – goes to ward on 1st week, develops plan before discharge, home visit 3 days post discharge • Jenson: Community based discharge planning – in reach, community nurse visits ward daily • Bonsack: Transitional case management –- a nurse, or a social worker was added to the treatment as usual procedure. Their role was not to replace the other care providers but to coordinate care provision and to represent the patient’s viewpoint. • Hampson: Community link team - to facilitate early discharge team-based service offering intensive support during the day.
Pharmacist Interventions	2	Abraham (2017), Shaw (2000)	• Abraham: Pharmacist consult intervention- psychiatrist has to order a pharmacist consult in the EHR for all LAI orders, hard copy of form sent to inpatient pharmacy and clinical pharmacist. Pharmacist has to approve LAI prescription before administered. Day of discharge injection clinic. Pharmacist led transitions in care program and medication delivery available prior to discharge. Following discharge continued treatment in outpatient clinic. • Shaw: Pharmacy discharge planning- baseline pharma needs assessment, information about medicines and then plan sent to community pharmacist
Intervention to prevent homelessness	2	Forchuk 2008, 2013	• Immediate assistance in accessing housing, assistance paying first month rent
(Psycho) educational	5	Kariel-Lauer (2000), Zheng (2005), Sato (2012), Khaleghparast (2013), Hegadus (2018)	• Kariel-Lauer: Re-entry group – short term group meetings, psychoeducational approach • Zheng: Family education- 8 h with service users, 36h with family in hospital, 2 h per month for 3 m post-discharge. Nurse with > 10 yr experience provided intervention. Purpose is to educate families about schizophrenia, treatment, teach skills to help families cope • Sato: Community re-entry program- discharge preparation programme – psychosocial preparation for long-term service users • Khaleghparast: Self-care training programme, delivered by nurses- 6 1 h sessions pre-discharge, 1 a fortnight post-discharge • Hegedus: Short transitional intervention in psychiatry – aims primarily to prepare patients for specific situations that could arise during the days immediately following discharge- cards with potential scenarios on
Needs-oriented discharge planning	2	Puschner (2011), Lin (2018)	• Puschner: Manualised needs led discharge planning and monitoring intervention with 2 intertwined sessions, 1 at discharge 1 3 months after. The intervention aimed at improving this communication (between primary and secondary) by means of information (needs assessment)-based standardised recommendations for outpatient treatment and monitoring of compliance with these recommendations. • Lin: Needs-orientated discharge planning for caregivers- nurses served as care coordinators and provided 6-step hospital discharge planning services to caregivers. Integrated therapeutic partnership, mental health education, and needs oriented services.
Whole Care Pathway Initiatives	2	Attfield (2017), Juven-Wetzler (2012)	• Attfield: Integrated care pathways- reducing unnecessary tests interventions and duplications- (ICPs), is a ‘multidisciplinary plan of care that provides detailed guidance for each stage in the care of a patient with a specific condition, over a given period of time’ • Juven-Wetzler: Continuation of care model– continuation of care by the same staff from the ward rather than outpatient referral
Multi-component interventions	3	Kidd (2016), Smelson (2010), Ghadiri Vasfi (2015),	• Kidd: Welcome Basket- 6 weeks- peer support- contact on wards prior to discharge and post, basket of items, environmental support • Smelson: Brief treatment engagement- 5 h per week of services in community- assertive community treatment using BCTI, peer support, dual recovery therapy • Ghadiri: Aftercare Services- 3 components, follow-up care (home visits or telephone), family psychoeducation, social skills training for patients
Discharge Checklist	1	Khanbahi (2018)	• Doctor's checklist as an aid memoir
Motivational Aftercare Planning	1	Kisely (2017)	• Motivational interviewing with advanced directives
Brief Care Management-	1	Taylor (2014)	• A brief interview that addresses goals and barriers to treatment which was administered by care managers of a managed behavioural health organization prior to the individuals’ discharge

### Critical time intervention

Critical time intervention (CTI) was the most frequently tested intervention found in this systematic search. CTI primarily aims to reduce homeless, in the ‘critical time’ following discharge from hospital. It is delivered by trained ‘CTI’ workers with small caseloads. It is community based and time limited (with gradually reduced contact). It involves 3 phases (1) Transition (2) Try-out (3) Transfer of Care. Five studies [9, 24–26] in the review reported on this intervention; which is primarily aimed at a particular population: those suffering homelessness and acute mental health hospitalisation. All of the studies were conducted in the USA. Two were randomised controlled trials (RCT) of 150 participants [9, 25], two non-randomised experimental design [24, 26] and one interviews with staff that deliver CTI (*n* = 12) [27]. Two studies found significant reduction in homelessness for those that received the intervention [9, 24]. Two studies also aimed to reduce readmission and found significant reductions in readmission rates in the CTI group compared to a control group [25, 26]. One study employed brief CTI [26]; which delivered the same intervention in a shorter time period. Finally, qualitative interviews found that CTI workers self-identified as ‘extra support’ and stressed the importance of three dyadic relationships to the success of the intervention: worker-client, worker-primary support and primary-support-client [27]. In summary, the studies reviewed suggest that CTI could be an effective method of reducing homelessness post-discharge and reducing early readmissions.

### Transitional discharge model/ transitional relationship model

The Transitional Discharge Model (TDM) also known as the Transitional Relationship Model, aims to increase continuity of care from hospital to community. Inpatient nurses work with service users until they establish a therapeutic relationship with their community worker. Support from peers (service users living successfully in the community) commencing prior to discharge and for up to 1 year after hospitalisation may be included in this package of support.. Three studies in this review tested this intervention, one was a large scale RCT; which found no significant differences in post-discharge costs or quality of life, but an unexpected finding of early discharge (on average 116 days earlier) [28]. A 25 participant randomised study found a significant reduction in readmissions [29]. The action-oriented research study highlighted the need to address inter-professional team working to improve staff uptake of the intervention [14]. Collectively, the results suggest that the TDM could be effective in reducing readmission and facilitating early discharge.

### Peer support

Peer support is when past service users use their own experiences to help current service users, primarily on a one-to-one basis, but it can exist in various forms. Three studies tested peer support as a distinct single intervention, although others included it as part of multi-component interventions. How peer support was delivered differed across studies, see Table 2. One evaluation study found a reduction in self-reports of readmission, functional and clinical recovery in 38 service users [30]. Another found benefits to service users and cost reductions for services [31]. However a small pilot RCT found no statistical differences in terms of hope or quality of life [32]. In summary, the studies testing peer support as an individual intervention used small sample sizes and reported heterogeneous outcomes, therefore it’s difficult to draw conclusions about its effectiveness.

### Contact-based interventions

The contact-based interventions were grouped together arbitrarily based on the provision of additional post-discharge contact with a professional beyond treatment as usual. Within this group there were various methods of making contact with service users. For example letters, telephone, face-to-face and video. The purpose of contact also varied across studies. Some aimed to reduce suicide, others to improve treatment adherence and one to reduce readmission. There were six contact based intervention studies in this review (see Table 2). Botha et al. aimed to reduce readmissions found no effect of a 90-day transitional care intervention (phone calls and home visits) [33]. Of the studies that aimed to reduce suicide, one found letters to recently discharged service users ineffective [6]. Whilst the other found only very early telephone follow-ups to be effective in a large-scale randomised controlled trial [5]. A small-scale RCT found intensive case management (weekly face-to-face contact and telephone calls) decreased suicidal ideation, increased service contact and satisfaction and improved relationships with professionals [34]. Two studies aimed to use technology to increase treatment adherence, one sent SMS messages and found a significant reduction in readmission and significant difference in treatment utilisation [35]. The other used an Multi-disciplinary team (MDT) videoconference with rural patients and reported greater treatment adherence in the intervention group [36]. In summary, as the interventions varied in terms of delivery and outcomes reported it’s difficult to draw conclusions. However, results collectively indicate that speed of follow-up contact is important in terms of suicide prevention and that contact-based interventions may not reduce readmission, but could be a useful mechanism for improving treatment adherence particularly in rural populations.

### Role-based interventions

Role-based interventions were defined by the introduction of a new role, position or job title in addition to treatment as usual. The specific tasks performed by each ‘role’ varied across the studies, see Table 2. Seven role-based intervention studies were included. One study introduced a discharge co-ordinator to improve communication between primary and secondary care but found no significant difference in outcomes (readmission, length of stay, satisfaction, mental health) in a large scale controlled study [37]. Similarly, one large scale RCT found no significant effect of a Post-Discharge Network Co-ordinator on readmission, social support, quality of life or mental health [38]. A case study of 3 patients from the original RCT revealed that this was likely to be due to the small, conflictual or unstable social networks of service users [39]. Two roles (community based discharge team and community links team) that focused on bridging the gap between inpatient and community care were found to be effective in either reducing readmission rates [40] or median length of stay [41]. The introduction of a case management role was found not to reduce readmissions [42]. However, a nursing discharge programme was found to improve medication adherence [43]. In summary, role-based interventions are introduced to address different aspects of the discharge process and, therefore comparison is difficult. However, all bar one of the role-based interventions, had no effect on readmission rates. This could indicate that readmission rates are not influenced by the introduction of new staff roles.

### Educational interventions

Educational interventions, focused on the delivery of training or education to service users or their families. Five educational interventions were included in the review, four of which were conducted in Asia. Four focus primarily on teaching various self-management techniques to service users, whilst one focuses on educating family members too. As these were educational interventions, the outcomes measured frequently concerned knowledge levels. Two studies reported a significant increase in knowledge about the psychological condition post intervention [44, 45]. Others aimed to use education to reduce readmission, two reported this effect [45, 46], whilst one reported no effect [47]. Educational interventions also showed some effect on reduction of symptoms, and treatment adherence [44–46]. One intervention taught service users how to cope and manage situations that may occur in the community. However no significant results were reported [48]. In summary, the outcomes measured in the educational interventions differ from those in the other interventions, with a greater focus on knowledge and behavioural outcomes, whilst there is an indication that psychoeducational interventions increase knowledge about ones condition, there is evidence to suggest that educational interventions may also improve some service-level outcomes such as readmission. However, the one study that reports behavioural/emotional outcomes reported no effect [48].

### Other interventions

There were some interventions included in the review that did not fit within the aforementioned primary categories, these groups had 3 or fewer studies, see Table 2. These were categorised as pharmacy interventions [11, 12] (medications focused interventions led by pharmacists), needs-orientated discharge planning [44, 49] (discharge planning interventions led by the needs of individuals), intervention to prevent homelessness (an intervention developed by Forchuk et al. [10, 50] focused only on homeless individuals), transitional care model (a nurse-based in home initiative) [51, 52], whole care pathway initiatives [53, 54] (that consider multiple agencies in the care pathway) and multi-component models [55–57] (using multiple interventions simultaneously). Despite studies reporting on these interventions they tended to be single instances and do not provide sufficient evidence for narrative synthesis on a categorical level.

### Variability of outcomes

Due to the vast differences in study design and populations, the outcomes measured varied considerably. Outcome was defined broadly when extracting the data to include anything that was measured or reported as a result of the intervention. Due to the differences in design, only RCTs reported primary and secondary clinical outcomes. Using this outcome definition, there were 69 unique outcomes reported across the studies. Whilst there were commonalities amongst some (readmission, length of stay, symptoms), many looked at specific outcomes in regards to a particular research question (addiction severity, concern about discharge, financial cost to system). Even those studies that reported the same outcomes measured them in different ways. For example, readmission was measured using various time frames, e.g. within a week, within 30 days, within a year. In addition, data were collected in various ways, e.g. interviews with service users or collecting hospital data. Table 3 shows the most commonly reported outcomes and the number of studies that reported each particular outcome.

**Table 3:** Number of studies that reported the most common outcomes

Outcome	No of Studies
Readmission	22
Length of stay	11
Mental health symptoms/psychopathology	10
Quality of life	7
Treatment adherence	5
Outpatient/appointment adherence	4
In housing	4
Global functioning	4
Service user satisfaction with discharge	4
Medication adherence	3
Depression	3
Knowledge about own condition	3
Service user satisfaction with treatment	3

### Theoretical assumptions

In order to consider the effectiveness of the interventions presented in this review, we need to first understand the underpinning theoretical assumptions of each intervention. Theory of change is not explicitly used in any of the reviewed studies, and many of the assumptions about the challenges associated with discharge are implicit within the design and evaluation of the intervention.

During the process of narrative synthesis, studies were clustered and grouped in multiple ways, one such way exemplified the threats to safety that the intervention aimed to solve and whether an effect was subsequently reported. As the outcomes were heterogeneous, it was difficult to directly compare outcomes, so instead we grouped the interventions in terms of the safety challenges they aimed to address, either implicitly or explicitly (see Additional file 5).

### Reducing readmission

The most common challenge that the interventions aimed to solve was readmission to an acute ward within a given short-term period, sometimes indicative of shortcomings in service provision [6, 12, 18, 25, 26, 28, 33, 36–38, 40, 41, 53, 54, 58–60]. Whilst some studies found evidence for a reduction in readmission due to the interventions [26, 28, 40], many failed to evidence this effect [6, 33, 36]. The studies that had an effect on readmission tended to focus on either education, therapeutic relationships or increased continuity of care [28, 61, 62]. Many of the successful interventions bridged the boundaries between ward and community by providing care from ward based professionals in the community [28, 54] or having community teams leading discharge planning on the wards [40]. Interventions that were successful in reducing readmission (primarily in Asian countries) had a psychoeducational focus [45, 46]. Other effective interventions were focused on a particular population, for example homeless individuals and managing financial/environmental challenges that these particular service users faced [25, 26].

More interventions were shown to have little effect on readmission than those that did. Some of these interventions shared commonalities with the successful interventions, for example the community link team, offering intensive support in the community during the day [41]. Contact-based interventions were particularly unsuccessful in terms of reducing readmission, for example videoconferencing [36], follow-up letters [6] and follow-up phone calls [33]. A few studies that considered care pathways from a staff-driven or service-level perspective were also not shown to be effective in terms of reducing readmissions [53, 58].

Many of the interventions that failed to reduce readmission, were arbitrarily categorised as role-based, e.g. psychiatric discharge co-ordinators [37], pharmacy discharge planners [12], community link team [41], post-discharge network co-ordinators [38]. This suggests that it may not be sufficient to introduce a new role as a single intervention. There is also evidence to suggest that care co-ordinating roles may result in high levels of stress and burnout [59]. Furthermore, issues that might lead to readmission are manifest across multiple dimensions, for example clinical, personal or social., Researchers are also increasingly questioning the validity of readmission outcome data, as better hospitals keep people alive, safe and cared for therefore multiple readmissions are in some cases an indication that a hospital is safer [18, 60].

A number of the interventions in this review that span boundaries have proven successful in terms of reducing readmission. There is also success in the psychoeducational interventions and those that focus on therapeutic relationships, indicating that tackling the personal and emotional elements of the care transition may be equally important when aiming to reduce psychiatric readmission. Whilst it is difficult to make any conclusions about effectiveness of interventions when the outcomes and geographical context are heterogeneous, the most promising results in terms of readmission involve reducing the epistemic, professional and physical boundaries between hospital and community. Therefore encouraging therapeutic relationships, education and empowerment of service users.

### Improving wellbeing and/or reducing symptoms

Many of the interventions that focused on the care transition inherently aim to improve wellbeing and reduce symptoms [6, 29, 35, 55]. The studies that report evidence of this have few commonalities, see Additional file 5. Whilst some contact based interventions show an effect [35] others show no effect [6]. Some interventions show an increase in quality of life but no change in symptoms [55], on the contrary, others report a reduction of symptoms but no increase in quality of life [29]. Perhaps this lack of clarity in the results could be a manifestation of using such subjective, difficult to measure outcomes that could be easily confounded by factors peripheral to the discharge intervention, rather than the effect of the transition intervention. Many of the interventions that focused on the transition between inpatient and community care inherently aimed to improve wellbeing and reduce symptoms. The studies that report evidence of this have few commonalities, see Additional file 5. Whilst some contact based interventions show an effect [35] others show none [6]. Some interventions show an increase in quality of life but no change in symptoms [55]. On the contrary, others report a reduction of symptoms but no increase in quality of life [29]. Perhaps this lack of clarity in the results could be a manifestation of using such subjective, difficult to measure outcomes that could easily be confounded by factors peripheral to the discharge intervention.

### Improving treatment and/or medication adherence

A number of studies aimed to improve treatment adherence [12, 35, 36, 40, 43, 50, 52, 56]. The few interventions that report success in improving treatment or medication adherence tend to be brief [50, 56], involve a co-ordinating agent [12, 43, 50] or use technology enhanced contact methods [35, 40]. Unlike readmission, the successful interventions that aim to increase treatment adherence tend to be role-based and some included a co-ordinating agent either a nurse [43, 52] or a pharmacist [12]. Similarly, whilst contact based interventions were less effective in reducing readmission, two contact based interventions improved treatment adherence [35, 36].

### Reducing homelessness

There were two interventions included in this review that focused on a sub-population within an acute ward, homeless individuals [10, 25–27, 63]. All of the interventions reported success in reducing homelessness [10, 25–27, 63]. The interventions studied by Forchuk and colleagues looked at financial assistance and support in accessing housing, essentially providing resources that service users might not otherwise have [10, 63]. Whereas the other intervention, CTI, focused on therapeutic relationships, and helping service users access services [25–27]. Both interventions focused primarily on homelessness but also reported benefits in terms of other outcomes like readmission.

### Reducing suicide

Only three studies in the review focused on reducing suicide post-discharge [5, 6, 34]. In the unsuccessful intervention 8 letters were sent to service users in the year after discharge, but this had no effect on suicide [6]. The two effective interventions focused either on early follow up post-discharge either by telephone [5] or consistent weekly face-to-face contact [34]. Indicating the immediacy and consistency may be key to interventions looking to solve this problem. This highlights that continuity of care with a human is more effective than indirect communication via letters as a means of reducing suicide post-discharge.

### Accelerating discharge

The interventions that are successful in reducing length of stay and accelerating discharge tend to have a systems or process level focus. For example integrated care pathways are evidenced to facilitate early discharge [53], as is introducing a community link team [41]. Whereas interventions that focus on a single element of the care pathway, or a single person seem less successful in solving this problem, for example the introduction of a team to co-ordinate discharge planning had little effect on length of stay [61, 64]. Similarly, introducing a single professional to address this challenge [37] or relying on the education of the service user [47], also proved unsuccessful. This could indicate that individual agents i.e. a single professional, team or service-user, are often disempowered within a multi-agency system, and therefore unable to generate meaningful change.

### Examination of facilitators and challenges/barriers

There were commonalities within the studies in terms of barriers and facilitators of effective implementation of interventions. All of which could be categorised as either staff level, service level, or service-user level, many sit within multiple categories. From a service level perspective, barriers were related to insufficient funding of services or interventions, ineffective information sharing and the effect of the physical location of services, particularly for rural community services [10, 12, 26, 50]. The structural effect was also a reported facilitator along with planning [65]. There were commonalities within the studies in terms of barriers and facilitators of effective implementation of interventions. All of which could be categorised as either staff level, service level, or service-user level and many sit within multiple categories. From a service level perspective, barriers were related to insufficient funding of services or interventions, ineffective information sharing and the effect of the physical location of services, particularly for rural community services [10, 12, 26, 61]. Effective planning was also a reported facilitator [64].

The effectiveness of an intervention was often highly dependent on the behaviours, opinions, affect and education of the staff delivering them. The willingness of staff to adapt and exhibit flexibility was key, as was providing staff with adequate training around the intervention [14, 28]. If staff worked in a multi-disciplinary manner, this was also considered facilitative to some interventions [52]. Having a ‘champion’ or staff member that advocates for the intervention was facilitative [14]. Staff were more responsive to interventions that reduced their workload or stress, as opposed to increasing it [14, 40, 41]. One staff level barrier was a lack of behaviour change in response to the intervention [28].

Barriers to successful intervention that were reported at a service-user level included behaviours that are often in opposition to recovery, for example substance-misuse, dependency on services or unstable social relationships [33, 39, 41]. Similarly, facilitators on a service-user level included behaviours or affect that are facilitative of recovery, such as a sense of belonging within community or community services, structured daily routine within the community and being a part of stable and structured social networks [30, 38, 40, 46, 51]. This does not indicate that the success of the intervention is dependent on the behaviour of the service users, but instead highlights the considerable effect of the complicated personal and social variables that surround mental health care transition interventions.

A number of facilitators and barriers transposed these distinct categories. From a barriers perspective, miscommunication, a lack of shared knowledge or accountability could be pejorative to intervention effectiveness on either a service, staff or service-user level or between groups [14]. Similarly, from a facilitator perspective comparable themes transcend the groups: communication and shared decision making within and between the groups was a key facilitator [52]. Connection amongst providers i.e. a supportive information sharing system, was also facilitative amongst staff groups and service providers [10]. Similarly, therapeutic relationships between staff and service users were considered facilitative [9, 27, 34]. Additional files 6 and 7 show visual depictions of barriers and facilitators.

### Assessing robustness

We used the Mixed Methods Appraisal Tool (MMAT) [20] to assess the quality of the studies included in the systematic review. It must be noted that a number of studies in this review had less than 20 participants and take an evaluative or pilot approach rather than a quantitative robust test of effectiveness. Additional file 3 outlines the quality assessment of each study. All of the studies included in the review met the screening criteria (used as a measure of minimum quality). The tool does not suggest researchers score the papers, however the table highlights the differences in study quality. As this systematic review does not make recommendations for the most effective intervention, the robustness of the studies is of lesser concern. Interestingly, many of the pilot studies found no or little effect of the interventions and it seems such interventions are rarely re-tested. For example, to our knowledge Walker et al., [37] is the only study to specifically test ‘discharge co-ordinators’ in relation to mental health, despite evidence that this method is effective in other patient populations and its inclusion in the UK National Institute for Health and Care Excellence (NICE) guideline regarding transitional care [66]. Some studies use a similar approach, such as care managers responsible for facilitating discharge [50], but the name ‘discharge co-ordinator’ has not been tested to our knowledge since. Hence, assumptions about the effectiveness of any particular intervention in this review are often based on the results of a small number of studies.

## Discussion

The studies included in the review are varied in terms of origin and design. Whilst this review uses a broad inclusion criteria to demonstrate the variability in challenges they aim to address, this means that there is variability of the baseline health systems in which the intervention is implemented, complicating comparison. For example, the interventions from Asia frequently reported higher effectiveness rates than those in the UK or USA, indicating that there are potentially cultural differences within complex systems that affect comparability of outcomes cross-culturally. This may include differences in the baseline or treatment as usual conditions. In the UK, for example, NICE guidelines recommend elements of some interventions as standard practice, (i.e. having a named staff member manage this discharge) [67]. Therefore, any effects of such interventions in the UK could be diminished within standard clinical practice.

This review highlights the different approaches that have been used internationally to tackle the varied challenges that discharge from an acute, inpatient mental health care poses. The variability of the interventions and the outcomes are likely to be a manifestation of the variation in how each research team interprets the problems associated with discharge. For example, those interventions that focus on pharmacist involvement, consider the active risk factor of medication non-adherence, whilst the contact-based, or whole system interventions articulate the problem of ineffective communication. Some researchers chose to only measure outcomes relevant to the specific problem they aim to address, whilst others do not articulate how the measures used indicate an improvement. Understanding the effective elements of interventions that address specific problems, would have greater advantages for healthcare professionals looking to improve practice or policy makers attempting to improve quality and safety at a service-level.

The interventions reviewed are spread across a spectrum ranging from addressing a single problem within a single agency with a single solution, to multiple solutions addressing multi-agency problems. Within which some interventions include multiple elements, i.e. 1) peer support 2) group meetings and 3) therapeutic relationship building. The notion that one intervention can solve a multitude of safety threats is also not evidenced in the wider care transitions literature [68, 67]. Hence, it’s difficult to assess the effect of each component of multi-stage interventions on each single problem, particularly without an explicit underlying theory of change.

Designated roles supporting the transition of service users from inpatient to community care was highlighted in a number studies included in this review. Care co-ordination has a long history within mental health services, for example in England and Wales the introduction of care co-ordinators stems from the Care Programme Approach in 1990 [69]. Assigned as the main point of contact for service users, care co-ordinators should facilitate care across agencies for an individual, including the transition from hospital to community. Care co-ordination is also an emergent concept in other areas of healthcare, particularly where individuals have complex needs. Whilst studies suggest that professionals working in co-ordination roles have high job satisfaction, they also experience high levels of stress and burnout [59, 70]. Therefore, when implementing new transitional roles consideration needs to be given to how they will fit with existing co-ordination roles and the support required by the individual undertaken them.

The heterogeneity in terms of outcome reporting made meaningful comparison of any interventions difficult. Even when interventions focused on a single solution, researchers measured select outcomes using different measurement tools. It is difficult therefore to assess the effectiveness of any single element with regards to any single outcome measure. This is in line with a systematic review of interventions that aimed to reduce readmission, whereby quantitative meta-synthesis could not be conducted [18]. This could also be exacerbated by the fact that the outcomes are arguably not indicative of the success of an intervention as they can be easily confounded by external variables. A recent report by The Kings Fund has questioned the validity of using clinical outcomes for a mental health population and recognised the importance of social and emotional outcomes [71].

Very few of the papers were explicit about the underlying theory of change. They often had unclear assumptions about what the nature of the problem was and how the interventions aimed to address it. This was further informed by the selection of the outcomes or measures used; which seemed in some cases to be pragmatic proxies rather than based upon a specific theory of change. For example, there is an emerging body of literature questioning the effectiveness of readmission as an outcome in mental health, as it only describes service use not clinical need [19, 72]. Research suggests that using a framework to guide improvement initiatives is beneficial, for example using the action-effect method (a systematic, structured approach to identify and articulate an improvement interventions theoretical assumption) [73].

In this paper we present a review of various discharge interventions that are not explicitly patient safety interventions, but that focus on improving quality and safety by addressing risk factors such as ineffective continuity of care or communication. All of the interventions aim to improve quality and safety, but are based upon limited understanding or articulation of what the quality and safety elements of healthcare are, nor are they informed by the safety literature. For example, they do not engage with patient safety literature that describes active and latent risk factors [74, 75], nor the literature around ‘systems-thinking’ approaches to managing risk [75]. Current thinking in the field of patient safety emphasises the contribution of upstream ‘latent factors’ in conditioning, exacerbating and enabling ‘active errors’ or mistakes in the organisation and delivery of care [74, 75]. These often involve local workplace and environmental factors, management pressures and organisational cultures. Such system factors are described as heightened at the point of discharge because care transitions tend to involve multiple sets of system factors interacting in the form of a complex system, as the patient moves across care domains [76]. Many of the threats to safety that are present in this time period are not explicit or directly visible in the working environment, as noted above they can be seen as latent risk factors whose impact on the continuity, quality and safety of care can be difficult to detect [74]. In relation to mental health care transitions, literature has outlined multiple systems-level risk factors in this time period, namely the lack of continuity of care and difficulties with communication between organisations and professionals [77–79] and many of the interventions implicitly or explicitly aimed to address one or more of these.

There are few unambiguous and conclusive findings from this review in terms of the effectiveness of interventions in addressing the distinct problems associated with discharge from acute mental healthcare settings, which is similar to other systematic reviews in this field [18, 72]. The synthesis suggests that the interventions that aim to reduce homelessness are generally effective [24, 26, 27, 63]. In particular, the review finds that these successful interventions either provide resources or psychosocial and/or therapeutic support in securing accommodation. This arguably indicates the importance of addressing a single risk factor with a single solution, ideally with an underpinning explicit theory of change. Similarly, with interventions that aim to improve treatment adherence, there seems to be some success in introducing a co-ordinating agent (assigned nurse, social worker or pharmacist) [12, 43, 50, 52] or technology enhanced contact methods [34, 35].

When considering the reduction of readmission, the most successful interventions aim to bridge the epistemic, professional and physical boundaries between hospital and community [14, 29, 61], either locating community staff on the ward, or ward staff in the community, increasing continuity of care or increasing knowledge of service users and families (see also [68]) [67].). Some examples of this are the Community-Based Discharge Planning [40] and the Transitional Discharge Model [29]. The commonality amongst interventions that successfully accelerated discharge is the use of a multi-agency, systems level approach to intervention [28, 41, 53], suggesting a systems-level approach is more successful than a single intervention (such as a new role) in accelerating discharge.

When considering the different types of interventions, there is some evidence that CTI is an effective method of reducing post-discharge homelessness. There is some evidence that the Transitional Discharge Model reduced readmission and facilitated earlier discharge. When peer support is used as a single intervention, there is very little evidence for effectiveness, this is probably due to the small evidence base of three studies and no effects reported in the only RCT. However, peer support has been used effectively as a component of a wider intervention that looks to increase continuity of care. Contact interventions varied in terms of format and scope in this review, however when addressing post-discharge suicide there some evidence that very early human contact could be effective. There is little evidence to suggest that contact-based interventions reduce readmissions, but some evidence for improvements in treatment adherence, particularly in rural populations. Similarly, role-based interventions were ineffective in reducing readmissions in all but one study, there is some evidence that introducing the correct roles could increase treatment adherence or reduce length of stay. The difference in reported success is likely due to the purpose of the roles. Roles that were introduced to address social/environmental factors were less successful than clinically focussed ones that aimed to increase treatment adherence. Boundary spanning roles (bridging hospital and the community) were also particularly successful. When considering the effect of interventions on readmission, it is important to re-consider the aforementioned emerging literature arguing against the effectiveness of readmission as outcome [19, 72].

Educational interventions seem highly successful in increasing knowledge outcomes in both service-users and care-givers. This increase in knowledge is also associated with subsequent effects in clinical and system-level outcomes such as readmission, symptom reduction and treatment adherence. The educational interventions predominantly originated from Asia and were uncommon in Europe and North America. However, the results could suggest that empowering service users and families/carers with knowledge rather than intervening only with staff and systems could be beneficial, beyond increased knowledge.

Overall the most effective interventions focus on addressing a single problem, they express explicitly the problem they aim to address and how success with be indicated by measurements. This is evident in the interventions that aim to address homelessness or increase knowledge of one’s condition. This could indicate that the precise specification of the intervention may be less important than how the intervention develops a better understanding of the problem, and hence uses a theory of change.

## Recommendations

In summary, to allow for a greater understanding of the elements of interventions that effectively reduce risk factors, a more structured approach to testing interventions is needed. This could be operationalised in multiple ways. First, by generating an agreed upon core outcome set to be used as standard in all future mental health discharge interventions (any unique outcomes would be used in addition to this). Second, more clarity is need in explicitly stating the problem (or latent risk factor) that an intervention aims to address (or each element in a multi-component intervention). This could explain or reduce the variability of effectiveness between similar interventions by providing more structure, transparency and means of comparison and subsequently advancement. In line with the majority of implementation research findings, the reviewed papers have very little underpinning theory (more specifically, theory of change) and articulation of what is needed within a complex system for the intervention to be successful [80]. Conceptualising these problems from a patient safety, systems-thinking perspective and with an explicit theory of change may make it easier to: 1) describe the specific problem the interventions aim to address; 2) understand the elements of an intervention that are effective to produce the desired intermediate or long term outcomes and c) understand what long term outcomes would indicate an effective intervention.

## Limitations

By utilising a less restrictive search strategy the outcomes reported are broad, and the aim of the studies varied. Thus making quantitative comparison difficult. Due to variances in outcomes reported, the quality of the studies used, cultural differences and the small number of within each intervention category no conclusive evidence can be drawn with regards to the effectiveness of any particular intervention. In an attempt to highlight and synthesise a breadth of interventions, the studies included in the review were not excluded due to risk of bias or quality, provided they met the basic screening questions in the MMAT. Due to budgetary constraints this review excluded papers that were not published in English and we acknowledge that this may have had an effect on the results of the synthesis and resulted in inclusion of papers that primarily represent English speaking cultures.

This review did not highlight sub-acute service models such as step-up or step-down services; which are increasingly common in the literature [86]. These are not specifically discharge interventions, but instead another service that often occupies the transitional gap. Future research should review the effects of sub-acute services in comparison to ‘discharge interventions’.

## Conclusions

There are numerous risk factors present in the chaotic, emotionally-charged period of discharge from an acute inpatient mental health ward. Heterogeneous interventions have been developed internationally in an attempt to solve some of these problems with variable success. Improving homogeneity of outcome reporting and applying theory of change to future research would allow better comparison of interventions.

## Supplementary information

**Additional file 1.** PRISMA checklist.

**Additional file 2.** Search terms.

**Additional file 3.** Mixed Methods Appraisal Tool.

**Additional file 4.** Diagram to outline the synthesis process.

**Additional file 5.** A diagram to show the differing safety challenges addressed by studies.

**Additional file 6.** Facilitators of interventions.

**Additional file 7.** Barriers that might affect interventions.

## Data Availability

The datasets used and/or analysed during the current study are available from the corresponding author on reasonable request.

## Abbreviations

BCTI: Brief Critical Time Intervention
CLT: Community Link Team
CoC: Continuity of Care
CTI: Critical Time Intervention
GP: General Practitioner
ICP: Integrated Care Pathway
LAI: Long Acting Injectable
LoS: Length of Stay
MDT: Multi-disciplinary team
MMAT: Mixed Methods Appraisal Tool
NHS: National Health Service
NICE: National Institute for Health and Care Excellence
QoL: Quality of Life
RCT: Randomised Controlled Trial
SMI: Serious Mental Illness
SMS: Short message service
SU: Service User
TAU: Treatment as Usual
TCM: Transitional Care Model
TDM: Transitional Discharge Model
TRM: Transitional Relationship Model
UK: United Kingdom
USA: United States of America
