# Supplementary material for: Interventions to improve discharge from acute adult mental health inpatient care to the community: systematic review and narrative synthesis
Source: BMC Health Serv Res. 2019 Nov 25;19:883. doi: 10.1186/s12913-019-4658-0 (PMC6876082; doi:10.1186/s12913-019-4658-0)
Supplement: Supplementary file 2 — Additional file 2. Search terms. [file 12913_2019_4658_MOESM2_ESM.docx]

Review Search Strategy

**PsycInfo**

1. discharge.mp. or exp FACILITY DISCHARGE/ or exp PSYCHIATRIC HOSPITAL DISCHARGE/ or exp HOSPITAL DISCHARGE/

2. client transfer/

3. transfer.mp.

4. handover.mp.

5. exp INTERVENTION/

6. discharge intervention.mp.

7. discharge planning.mp.

8. discharge coordinator.mp.

9. discharge summary.mp.

10. exp mental disorders/

11. mental* disorder*.mp.

12. exp Mental Health/

13. mental* health*.mp.

14. mental illness*.mp.

15. exp Schizophrenia/ or schizophr*.mp.

16. eating disorder.mp. or exp Eating Disorders/

17. exp Major Depression/

18. depress*.mp.

19. exp Affective Disorders/

20. mood disorder.mp.

21. exp BIPOLAR DISORDER/ or bipolar.mp.

22. suicid*.mp.

23. anorex*.mp.

24. bulim*.mp.

25. exp Posttraumatic Stress Disorder/

26. PTSD.mp.

27. 10 or 11 or 12 or 13 or 14 or 15 or 16 or 17 or 18 or 19 or 20 or 21 or 22 or 23 or 24 or 25 or 26

28. 1 or 2 or 3 or 4

29. 5 or 6 or 7 or 8 or 9

30. 27 and 28 and 29

Limit 2000-2018

**Number of papers returned: 686**

**Date 04/5/2018**

**MEDLINE**

**As above**

**Number of papers returned : 253**

**09/05/2018**

**EMBASE**

**As above**

**Number of papers returned: 611**

**09/05/2018**

**HMIC**

**As above**

**Number of papers returned: 39**

**04/05/2018**

**IBSS**

noft(discharge OR transfer OR handover OR psychiatric hospital discharge OR hospital discharge ) AND noft(intervention OR discharge intervention OR discharge planning OR discharge coordinator OR discharge summary) AND noft(mental health OR mental illness OR psychiatric disorder OR mental disorder OR anxi* OR depress* OR PTSD OR anorex* OR bullim* OR schiz* OR bipolar OR "Eating Disorder" OR "mood disorder" OR "affective disorder" OR suicid*)

NOFT= everything except full text

Limited to peer reviewed

Limited to 2000-2018

Results 72

Date 09/05/2018

**Cochrane Library**

**Trials**

Title: **(discharge OR transfer OR handover OR "psychiatric hospital discharge" OR "hospital discharge" )**

**AND TITLE-ABS-KEY (intervention OR "discharge intervention" OR "discharge planning" OR "discharge coordinator" OR "discharge summary") AND ("mental health" OR "mental illness" OR "psychiatric disorder" OR "mental disorder" OR anxi* OR depress* OR PTSD OR anorex* OR bullim* OR schiz* OR bipolar OR "Eating Disorder" OR "mood disorder" OR "affective disorder" OR suicid*)**

**Excluded before 2000**

Results: 123

Date 09/05/2018

**Scopus**

## ( TITLE ( ( discharge  OR  transfer  OR  handover  OR  "psychiatric hospital discharge"  OR  "hospital discharge" )  AND  ( intervention  OR  "discharge intervention"  OR  "discharge planning"  OR  "discharge coordinator"  OR  "discharge summary" ) )  AND  TITLE-ABS-KEY ( ( intervention  OR  "discharge intervention"  OR  "discharge planning"  OR  "discharge coordinator"  OR  "discharge summary" ) )  AND  TITLE-ABS-KEY ( ( "mental health"  OR  "mental illness"  OR  "psychiatric disorder"  OR  "mental disorder"  OR  anxi*  OR  depress*  OR  ptsd  OR  anorex*  OR  bullim*  OR  schiz*  OR  bipolar  OR  "Eating Disorder"  OR  "mood disorder"  OR  "affective disorder"  OR  suicid* ) ) )

## Excluded earlier than 2000

**Results 101**

**Date 09/05/2018**

**Web of Science**

**TITLE**:((discharge OR transfer OR handover OR "psychiatric hospital discharge" OR "hospital discharge" )) *AND* **TOPIC:**((intervention OR "discharge intervention" OR "discharge planning" OR "discharge coordinator" OR "discharge summary")) *AND***TOPIC:** (("mental health" OR "mental illness" OR "psychiatric disorder" OR "mental disorder" OR anxi* OR depress* OR PTSD OR anorex* OR bullim* OR schiz* OR bipolar OR "Eating Disorder" OR "mood disorder" OR "affective disorder" OR suicid*))

**Timespan:** 2000-2018. **Indexes:** SCI-EXPANDED, SSCI, A&HCI, CPCI-S, CPCI-SSH, ESCI.

**Results 352**

**Date 09/05/2018**

**CINAHL**

## ( TITLE ( ( discharge  OR  transfer  OR  handover  OR  "psychiatric hospital discharge"  OR  "hospital discharge" )  AND  ( intervention  OR  "discharge intervention"  OR  "discharge planning"  OR  "discharge coordinator"  OR  "discharge summary" ) )  AND  TITLE-ABS-KEY ( ( intervention  OR  "discharge intervention"  OR  "discharge planning"  OR  "discharge coordinator"  OR  "discharge summary" ) )  AND  TITLE-ABS-KEY ( ( "mental health"  OR  "mental illness"  OR  "psychiatric disorder"  OR  "mental disorder"  OR  anxi*  OR  depress*  OR  ptsd  OR  anorex*  OR  bullim*  OR  schiz*  OR  bipolar  OR  "Eating Disorder"  OR  "mood disorder"  OR  "affective disorder"  OR  suicid* ) ) )

**Date restricted 2000-2018**

**Results 142**

**Date 09/5/2018**

**ASSIA**

[**title(discharge OR transfer OR handover OR psychiatric hospital discharge OR hospital discharge) AND noft(intervention OR discharge intervention OR discharge planning OR discharge coordinator OR discharge summary) AND noft(mental health OR mental illness OR psychiatric disorder OR mental disorder OR anxi* OR depress* OR pood OR anorex* OR bullim* OR schiz* OR bipolar OR "Eating Disorder" OR "mood disorder" OR "affective disorder" OR suicid*)**](https://search-proquest-com.ezproxy.nottingham.ac.uk/results.displayspellingsuggestions_0:dospellingsearch?site=assia&t:ac=472C71565A0B4E9FPQ/1)

Limited to peer reviewed and 2000+

**Results 90**

**09/05/2018**

**Colation and Screening**

| **Assia** | **90** |
| --- | --- |
| **Cinahl** | **142** |
| **Cochrane** | **123** |
| **embase** | **611** |
| **hmic** | **39** |
| **ibss** | **72** |
| **medline** | **253** |
| **psycinfo** | **687** |
| **scopus** | **101** |
| **wos** | **352** |
| **Total number of hits** | **2482** |
| **Total unique papers** | **1630** |
