# Supplementary figures and images for: Interventions to improve discharge from acute adult mental health inpatient care to the community: systematic review and narrative synthesis
Source: BMC Health Serv Res. 2019 Nov 25;19:883. doi: 10.1186/s12913-019-4658-0 (PMC6876082; doi:10.1186/s12913-019-4658-0)

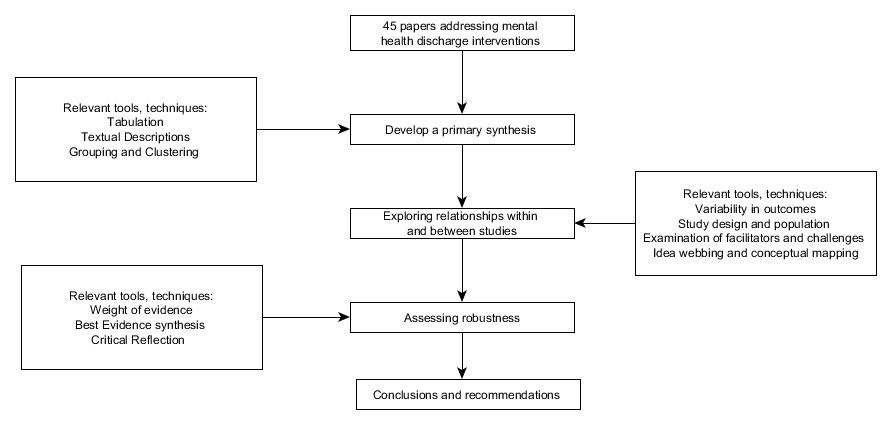

Supplement: Supplementary file 4 — Additional file 4. Diagram to outline the synthesis process. [file 12913_2019_4658_MOESM4_ESM.jpg]

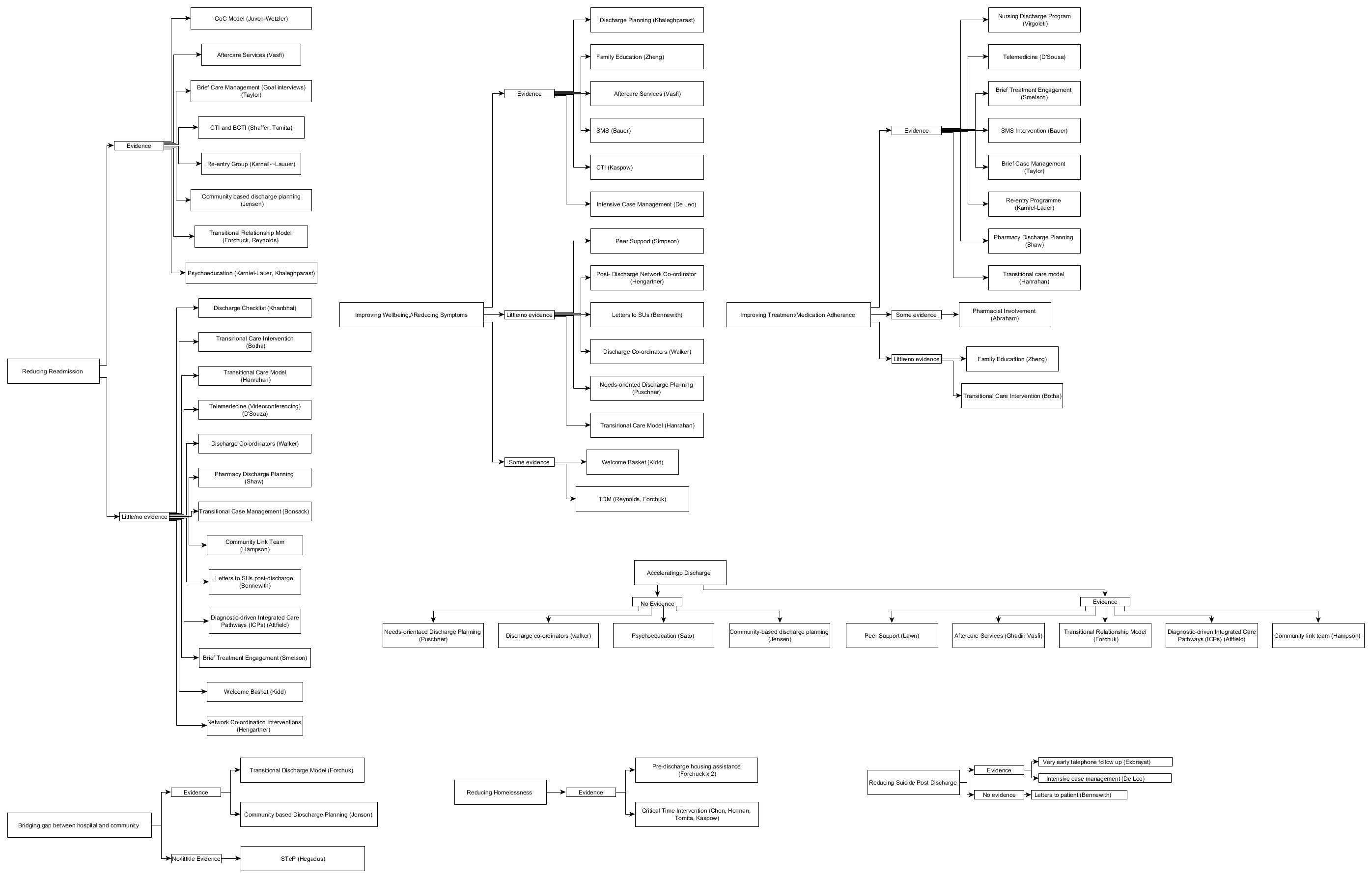

Supplement: Supplementary file 5 — Additional file 5. A diagram to show the differing safety challenges addressed by studies. [file 12913_2019_4658_MOESM5_ESM.jpg]

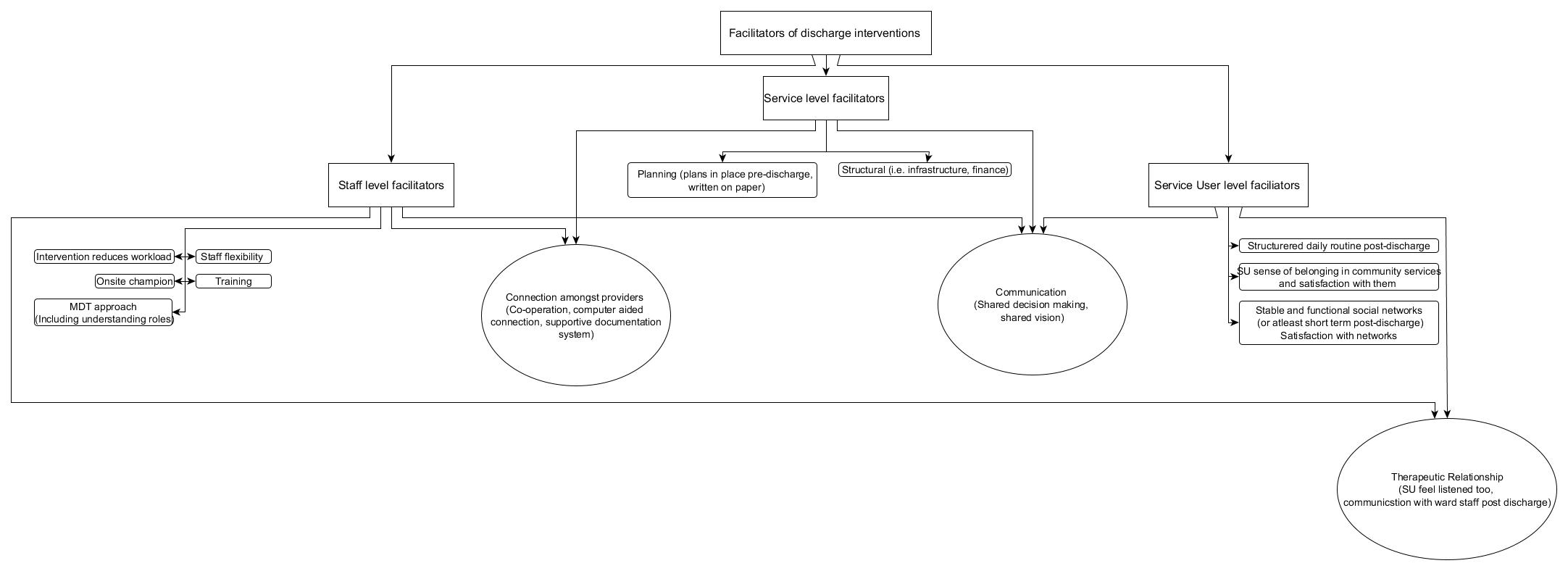

Supplement: Supplementary file 6 — Additional file 6. Facilitators of interventions. [file 12913_2019_4658_MOESM6_ESM.jpg]

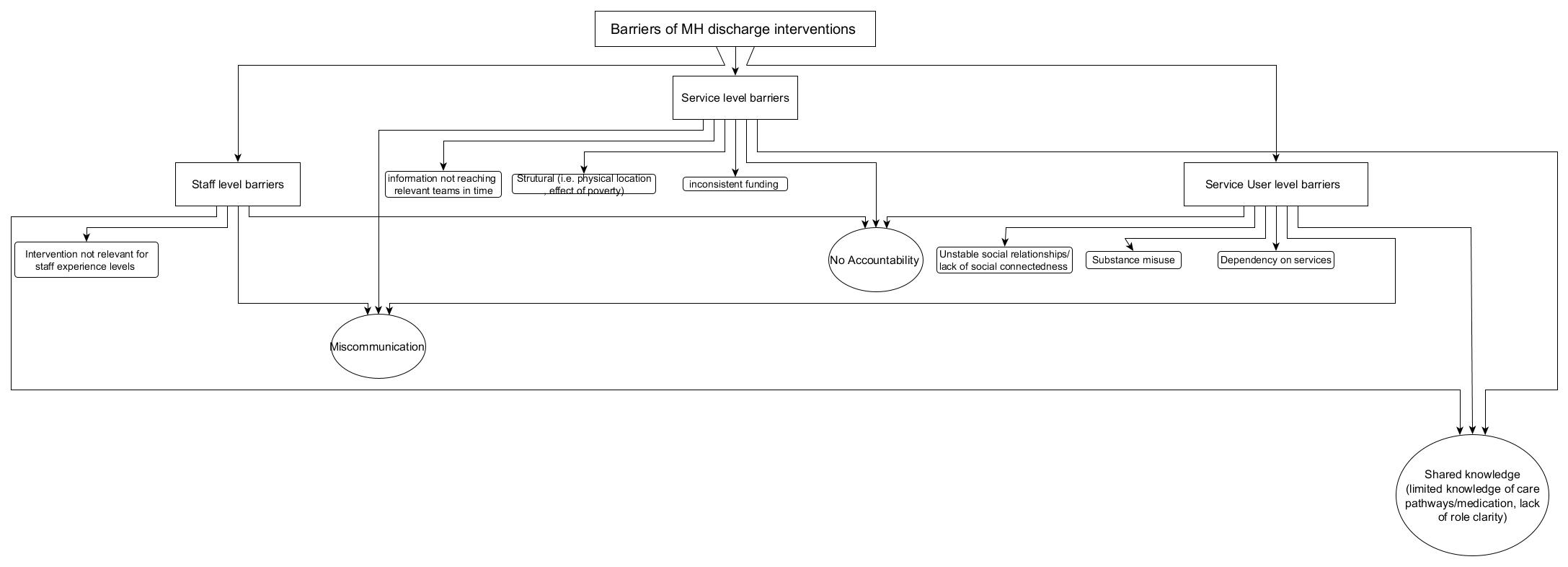

Supplement: Supplementary file 7 — Additional file 7. Barriers that might affect interventions. [file 12913_2019_4658_MOESM7_ESM.jpg]
